# Supplementary material for: Fungal effector SIB1 of Colletotrichum orbiculare has unique structural features and can suppress plant immunity in Nicotiana benthamiana
Source: J Biol Chem. 2021 Oct 29;297(6):101370. doi: 10.1016/j.jbc.2021.101370 (PMC8633582; doi:10.1016/j.jbc.2021.101370)
Supplement: Supplemental Figures S1–S5 and Tables S1, S2 [file mmc1.pdf]

Table S1. Top 100 genes of putative secreted proteins highly expressed on 1 dpi of *C. orbiculare* on *N. benthamiana* (*Nb*)

Candidates	Analyzed genes	Cob ID	Amino acid length	1 dpi in *Nb* RPKM means	3 dpi in *Nb* RPKM means	7 dpi in *Nb* RPKM means	Conidia RPKM means	Vegetative hypha RPKM means
CE1		Cob_v001234.t1	83	3,678.29	8,763.85	2,677.76	56.14	4,988.52
		Cob_v013223.t1	109	3,468.15	98.99	0.91	0.03	1.50
CE2		Cob_v005496.t1	82	3,109.53	9,016.82	4,023.70	71.38	29,651.22
CE3		Cob_v009911.t1	185	3,085.65	161.15	1,315.67	11,262.04	0.06
		Cob_v001246.t1	68	2,808.95	1,266.92	676.91	22.92	6.13
CE4		Cob_v010277.t1	306	2,410.85	4,195.09	2,931.33	44.07	13,370.39
CE5(*SIB2*)		Cob_v007489.t1	99	2,244.45	143.91	65.23	0.36	0.03
		Cob_v001026.t1	257	2,218.84	28.30	4,212.83	8,051.11	4.05
		Cob_v007230.t1	242	2,037.93	209.38	10.71	5.35	1.01
		Cob_v009935.t1	139	1,972.29	202.87	1.56	0.65	24.36
		Cob_v004706.t1	230	1,838.48	143.39	84.89	1.16	0.05
	CAD1	Cob_v002141.t1	101	1,668.50	2,105.92	468.35	88.58	71.76
		Cob_v008313.t1	279	1,444.59	49.56	628.35	7,704.97	0.89
		Cob_v011048.t1	196	1,409.90	373.83	191.31	71.14	92.43
		Cob_v011669.t1	715	1,399.85	890.82	560.28	60.98	1,081.33
CE6		Cob_v007191.t1	143	1,316.62	446.77	539.15	385.69	511.65
		Cob_v000444.t1	275	1,271.60	152.47	2.51	1.96	0.91
		Cob_v011375.t1	369	1,167.54	99.15	557.88	1,619.22	4.74
		Cob_v010667.t1	193	1,135.31	214.30	28.94	16.03	17.58
		Cob_v002919.t1	659	1,130.45	87.54	13.49	19.79	0.44
		Cob_v005023.t1	110	1,128.81	7,422.17	1,313.18	628.08	1,921.27
		Cob_v013109.t1	230	1,074.10	141.64	9.54	2.23	1.81
		Cob_v004771.t1	218	1,050.50	58.74	13.51	18.64	0.48
		Cob_v010981.t1	77	1,034.64	840.80	190.79	6.14	668.88
		Cob_v010633.t1	329	1,009.32	40.43	4.49	10.78	0.14
CE7(*SIB1*)		Cob_v007924.t1	70	981.60	592.87	283.79	12.33	3.71
		Cob_v008434.t1	402	961.28	268.90	563.86	364.14	16.58
		Cob_v001199.t1	185	922.23	109.29	14.94	34.20	8.90
		Cob_v011243.t1	151	893.62	27.96	448.06	2,044.57	5.98
		Cob_v011037.t1	407	881.99	100.27	1.15	0.17	0.05
		Cob_v011085.t1	168	873.58	14.80	0.34	0.14	0.00
		Cob_v011280.t1	330	870.84	58.51	0.09	0.05	0.05
		Cob_v000672.t1	198	870.11	737.42	631.30	127.95	643.05
CE8		Cob_v012447.t1	161	858.93	195.87	48.33	10.25	184.16
		Cob_v006053.t1	339	812.24	123.46	29.49	25.74	8.47
		Cob_v011399.t1	415	785.84	342.87	253.95	467.35	419.56
		Cob_v011639.t1	187	766.62	463.80	444.85	26.47	257.33
		Cob_v008170.t1	572	733.15	99.38	1,162.17	594.45	0.04
		Cob_v013099.t1	166	722.14	80.64	6.33	0.22	0.75
		Cob_v005847.t1	535	720.77	430.17	869.81	1,292.39	1,546.01
		Cob_v009518.t1	153	696.07	16.91	0.16	0.04	4.01
		Cob_v008050.t1	192	681.69	131.63	74.65	11.42	0.09
		Cob_v004735.t1	97	680.09	2,533.17	464.97	904.73	764.51
	NIS1	Cob_v001680.t1	162	673.37	264.23	84.37	554.56	227.45
		Cob_v007540.t1	335	662.49	21.86	385.75	3,326.02	1.83
		Cob_v008826.t1	81	651.36	51.69	0.75	0.04	0.13
		Cob_v003220.t1	227	649.95	1,476.68	956.38	90.66	1,265.92
		Cob_v005550.t1	333	643.20	15.12	213.88	1,794.89	8.67
		Cob_v007094.t1	122	625.11	130.78	55.51	27.92	1,068.75
		Cob_v000074.t1	113	624.17	465.30	83.27	3.43	13.92
		Cob_v001644.t1	87	620.58	2,049.03	716.97	52.74	1,670.22
		Cob_v005790.t1	509	611.51	17.84	75.96	2,527.52	0.05
		Cob_v012430.t1	258	585.68	20.34	314.06	2,469.30	0.54
		Cob_v001702.t1	667	573.72	1,501.00	511.51	232.54	1,278.44
		Cob_v007288.t1	497	560.23	58.22	9.45	19.33	3.43
		Cob_v001383.t1	126	557.63	513.65	485.98	91.89	222.67
		Cob_v012956.t1	233	550.77	18.00	211.56	442.02	0.01
		Cob_v000987.t1	158	550.26	28.99	658.76	933.98	0.33
		Cob_v003202.t1	801	549.69	77.74	118.76	2,464.18	1.75
		Cob_v009705.t1	134	539.14	78.86	43.20	42.04	7.69
		Cob_v000983.t1	218	536.62	708.52	2,332.27	150.49	3,241.83
		Cob_v012875.t1	341	530.83	166.50	645.34	100.90	124.78
		Cob_v004588.t1	267	525.40	610.14	457.07	381.35	530.37
		Cob_v001452.t1	88	522.33	56.28	0.16	0.00	0.08
		Cob_v001429.t1	465	502.19	1,271.57	446.16	14.77	3,525.54
		Cob_v012258.t1	80	495.40	1,652.67	151.33	3.60	2,858.80
		Cob_v012139.t1	324	491.00	701.92	457.35	6.98	8.38
		Cob_v012955.t1	110	487.12	137.82	609.33	478.29	306.36
		Cob_v001442.t1	225	478.12	36.19	20.84	0.23	0.17
		Cob_v006870.t1	319	474.47	89.76	164.61	1,229.17	28.63
		Cob_v010972.t1	552	472.16	552.88	1,042.23	468.16	1,359.79
		Cob_v007780.t1	450	468.99	50.47	180.51	1,849.89	2.99
		Cob_v011394.t1	258	466.97	23.36	116.57	3,231.73	0.84
		Cob_v010839.t1	249	462.78	38.80	28.25	10.83	6.12
		Cob_v001587.t1	228	449.43	150.93	54.76	13.75	6.69
		Cob_v000175.t1	141	441.46	606.29	740.54	580.11	1,034.52
		Cob_v000340.t1	164	440.04	483.23	528.43	539.22	1,226.58
		Cob_v008040.t1	75	436.94	148.39	6.38	0.90	17.83
		Cob_v000963.t1	254	426.48	1,820.65	1,655.97	115.30	5,803.44
		Cob_v009987.t1	260	419.67	46.87	2.65	1.35	0.39
		Cob_v008911.t1	516	413.51	74.20	37.68	42.07	15.21
		Cob_v008083.t1	480	411.79	1,563.42	740.01	0.83	410.54
		Cob_v003303.t1	318	407.52	35.69	15.70	4.36	2.14
		Cob_v001651.t1	186	400.68	20.43	10.87	0.88	0.04
		Cob_v003804.t1	91	396.60	2,101.15	110.27	5.17	29.13
		Cob_v009986.t1	506	394.87	43.00	2.78	2.02	0.04
		Cob_v003540.t1	786	381.50	93.06	127.24	35.14	18.09
		Cob_v010108.t1	574	379.67	639.45	253.09	11.72	3,421.70
		Cob_v008435.t1	509	372.68	199.26	236.35	170.66	11.40
		Cob_v011325.t1	435	370.55	560.30	566.91	208.54	1,384.57
		Cob_v001607.t1	562	367.54	53.57	3.15	67.22	0.24
		Cob_v007299.t1	284	363.61	271.38	72.23	13.63	14.72
		Cob_v005981.t1	224	363.07	81.18	35.04	502.66	47.51
		Cob_v001537.t1	509	356.44	49.44	124.69	777.95	52.43
		Cob_v003509.t1	85	350.01	875.34	550.23	33.09	59.53
		Cob_v007097.t1	547	348.48	131.37	128.19	2.24	542.73
		Cob_v005084.t1	150	347.67	617.83	321.46	372.99	479.17
		Cob_v009433.t1	188	344.77	60.14	10.51	6.17	2.67
		Cob_v001304.t1	210	330.41	356.41	1,031.42	1,582.15	533.86
		Cob_v008205.t1	193	329.59	41.51	1.54	0.10	0.63

Table S2. List of synthetic DNA used for this study.

Primer name	Sequence (5' to 3')	Restriction enzyme site
A. Primers for transient expression in *N. benthamiana*		
35S_CE1_Fw	AGGCCTACGGGGATCCAAGGAGATATAACAATGCAGTTCTTCAAG	*Bam*HI
35S_CE1_Rv	GGAATTCCGGGGATCCTTAGCAGCTCCAGTAGGG	*Bam*HI
35S_CE2_Fw	AGGCCTACGGGGATCCAAGGAGATATAACAATGCAGTTCTTCAA	*Bam*HI
35S_CE2_Rv	GGAATTCCGGGGATCCTTAAGGGCAGCTCCAGTAG	*Bam*HI
35S_CE3_Fw	AGGCCTACGGGGATCCAAGGAGATATAACAATGCATTTTTCCAGCA	*Bam*HI
35S_CE3_Rv	GGAATTCCGGGGATCCCTACGTGAAGGTCTTGACC	*Bam*HI
35S_CE4_Fw	AGGCCTACGGGGATCCAAGGAGATATAACAATGTTCGCTTCTACC	*Bam*HI
35S_CE4_Rv	GGAATTCCGGGGATCCCTAAGGAGACAGGGAGATG	*Bam*HI
35S_CE5_Fw (*SIB2*)	AGGCCTACGGGGATCCAAGGAGATATAACAATGGTTTCCTTCAAG	*Bam*HI
35S_CE5_Rv (*SIB2*)	GGAATTCCGGGGATCCCTACCAGTTGCCGTTGTTG	*Bam*HI
35S_CE6_Fw	AGGCCTACGGGGATCCAAGGAGATATAACAATGCAGTTCCTCAA	*Bam*HI
35S_CE6_Rv	GGAATTCCGGGGATCCTTACTCGAGGTCGAAGGAG	*Bam*HI
35S_CE7_Fw (*SIB1*)	AGGCCTACGGGGATCCAAGGAGATATAACAATGCAGCTCACCAAGCTCTTT	*Bam*HI
35S_CE7_Rv (*SIB1*)	GGAATTCCGGGGATCCTTACTTAGGGCAGCTTGCA	*Bam*HI
35S_CE8_Fw	AGGCCTACGGGGATCCAAGGAGATATAACAATGCGCTTCTCTAT	*Bam*HI
35S_CE8_Rv	GGAATTCCGGGGATCCTCACGACGCGCCGGCCTC	*Bam*HI
35S_SIB1_Fw	CGGGATCCAAGGAGATATAACAATGCAGCTCACCAAGCTCTTT	*Bam*HI
35S_SIB1-HA_Rv	CGGGATCCTTAAGCGTAATCTGGAACATCGTATGGGTACTTAGGGCAGCTTGCAG	*Bam*HI
35S_eGFP_Fw	CGGGATCCAAGGAGATATAACAATGGTGAGCAAGGGCGAGGAG	*Bam*HI
35S_eGFP-HA_Rv	CGGGATCCTCAAGCGTAATCTGGAACATCGTATGGGTACTTGTACAGCTC	*Bam*HI
B. Primers for gene disruption		
CoLIG5SN2	CAGGTTGCGGCCGCAAGGAGCCAAGTCGAG	*Not* I
CoLIG5ASN2	ATAAGAATGCGGCCGCATGGCACTGGAGTGTCCGTACTCT	*Not* I
CoLIG3SA5	GAACGGGCCCCTTTCGGGACACTGAAGACG	*Apa* I
CoLIG3ASA5	CGCAAGGGGCCCGAGCCCGTGGAGTACTACAACAACCTCC	*Apa* I
GENAS1B	GGCGGATCCTCAGAAGAACTCGTCAAGAAG	*Bam*HI
GENS1X	GCCCCTCTAGACAGACACAATGATTGAACAGATGGATTGC	*Xba* I
Co5-Jcheck3	GCATGTGTGTGACCCTATCC	—
J-check-CoLIG3AS	GTTTGACCTTCAACCAATCG	—
SIB1_Up_Fw	GCGAATTGGGTACCGGGCCCTCATCAGGCCTTCAAATTTC	*Apa* I
SIB1_Up_Rv	GTCGACTCGAGGGGGGGCCCTTTGGCTGTTTGTGTCTGAT	*Apa* I
SIB1_Down_Fw	GCTTGATATCGAATTCGAGGTGTGGAAGGCGAGG	*Eco*RI
SIB1_Down_Rv	CGGGCTGCAGGAATTCAGTGACGATGTATGCAGCG	*Eco*RI
SIB1_col_F	CCAAATAGATGACGTTGAACC	—
SIB1_col_R	TACAAGCTCACATTTACCGAG	—
C. Primers for RT-qPCR		
CoACT1_F	CTCGTTATCGACAATGGTTC	—
CoACT1_R	GAGTCCTTCTGACCCATACC	—
SIB1_qRT_F	CCAAGCTCTTTGTTGCGACC	—
SIB1_qRT_R	TTTGACGCCGCTAGTGTTCT	—
D. Artificial *SIB1* for tobacco BY-2 expression (Optimized for *Nicotiana tabaccum*)		
SP-SIB1	AAAGTTTAAACAATGAAAACTAATCTCTTCTTATTCCTTATCTTCTCCTTACTTCTCTCTTTGTCCTCCGCTCAAGAA<br>GGAAAATGTACTGCTAAGGGTGAATGCCAAGAGAACACTTCTGGAGTTAAATTGTTTTGTACTTCTGGTTCATG<br>CGCTAAGAAAGAGGGTCAGGCTTGCACTAGAAATGGTCCAGGTTCATCCAACTCCGCATCATGTCCTAAATAAT<br>AAGGTAACCAAA<br>Underline : Signal peptide sequence of Arabidopsis chitinase (NCBI Accession No.: AAK96819)	*Pme* I, *Bst* EII

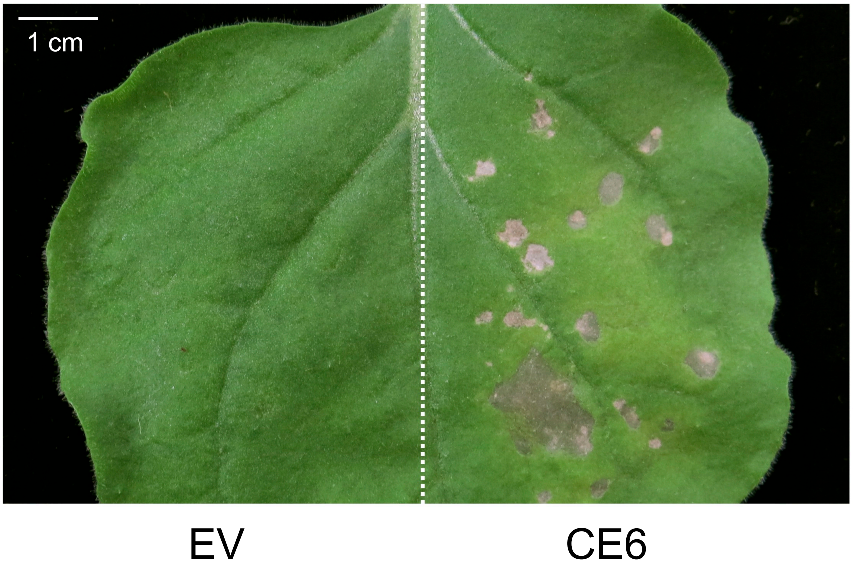

Figure S1. *Agrobacterium*-mediated expression of CE6 caused cell death in *N. benthamiana*. *Agrobacterium* carrying the empty vector (EV) or pBICP35-CE6 was infiltrated into *N. benthamiana*. Lesion development was observed at 7 days after agroinfiltration. Similar results were obtained from one additional experiment.

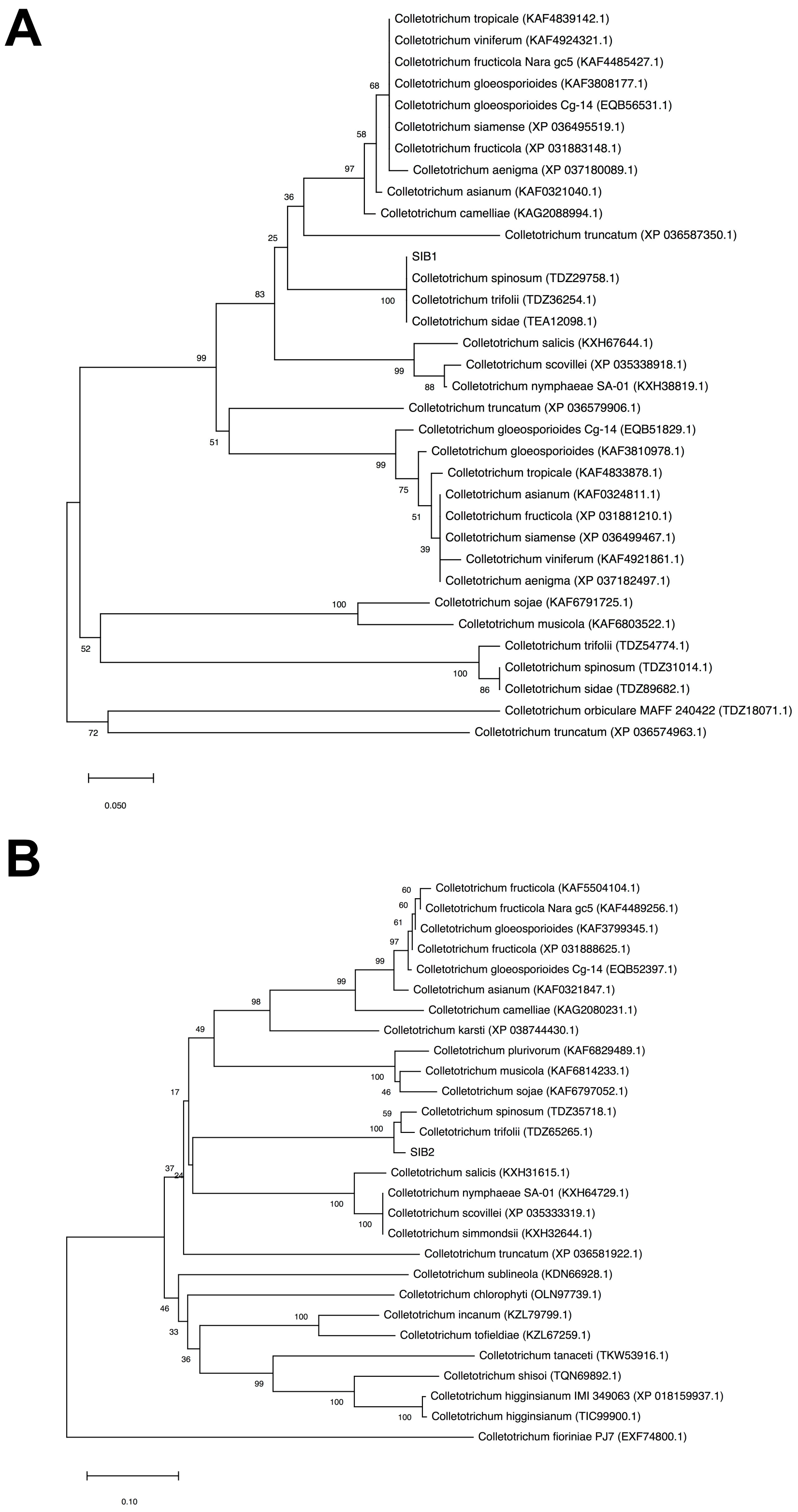

Figure S2. Phylogenetic analysis of SIB1 and SIB2. Phylogenetic trees were constructed using the neighbor-joining method in MEGAX. The numbers on each node represent the bootstrap values. The scale bar indicates the evolutionary distance in amino acid substitution per site.

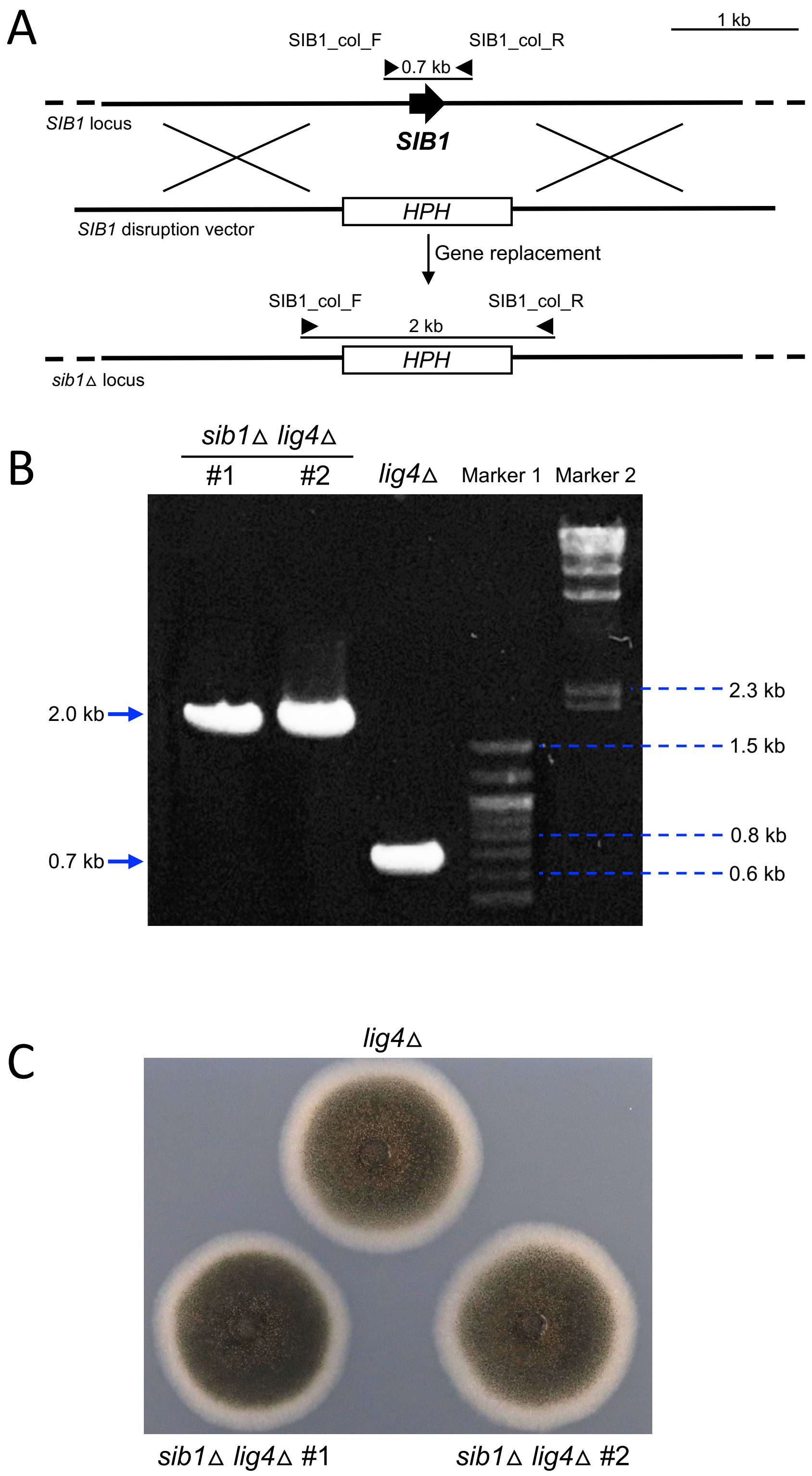

Figure S3. Gene disruption of *SIB1* and colony growth of the *sib1*Δ strains. (A) Using the *SIB1* gene disruption vector containing a hygromycin phosphotransferase gene (*HPH*) cassette, *SIB1* was replaced by the *HPH* cassette in *C. orbiculare*. (B) Genomic PCR analysis of *SIB1* gene disruption mutants (lanes 1 and 2) and *C. orbiculare* parental strain (lane 3) with primers SIB1_col_F and SIB1_col_R. (C) Colony growth of the *sib1*Δ *lig4*Δ strains and *lig4*Δ strain of *C. orbiculare* grown on PDA for 7 days.

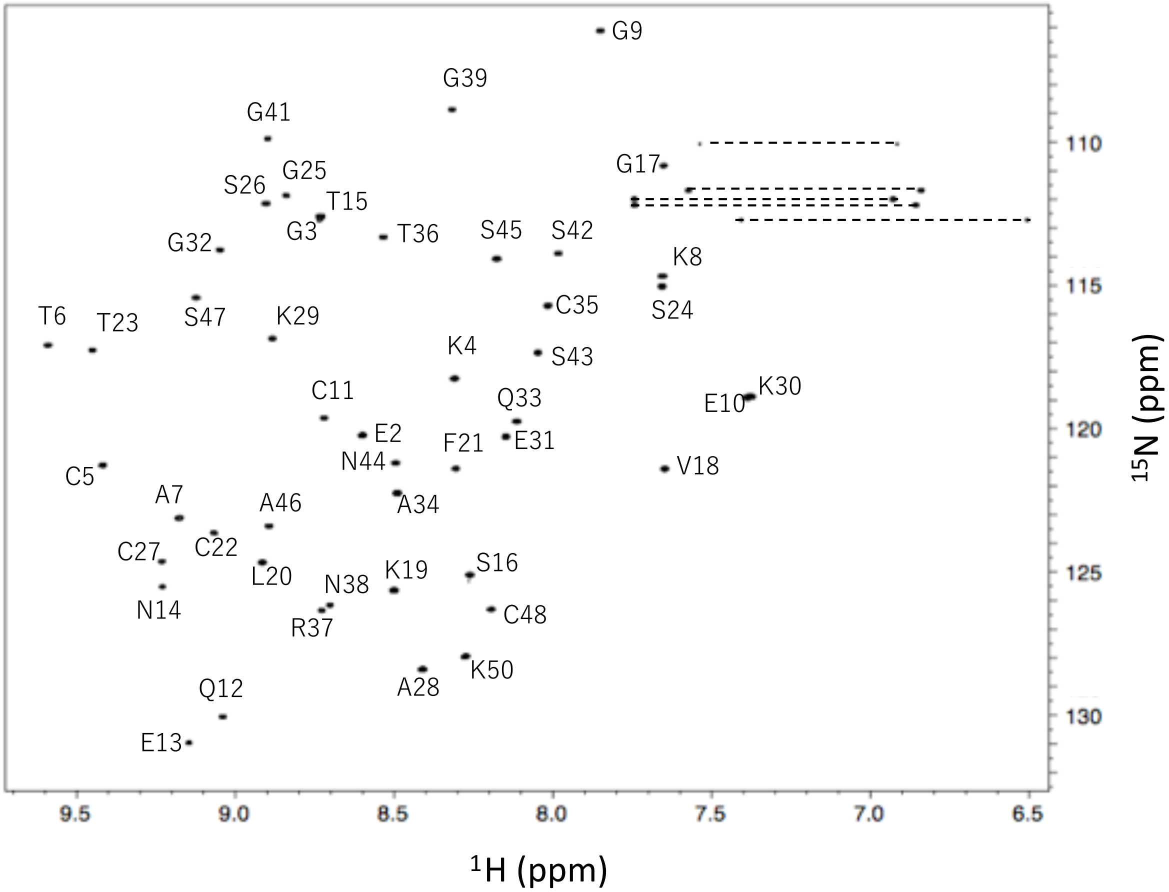

Figure S4. ^{1}H-^{15}N HSQC of SIB1. The assigned amino acid type and residue number are shown for each peak. Dashed lines indicate the pairs of Gln and Asn side chain resonances.

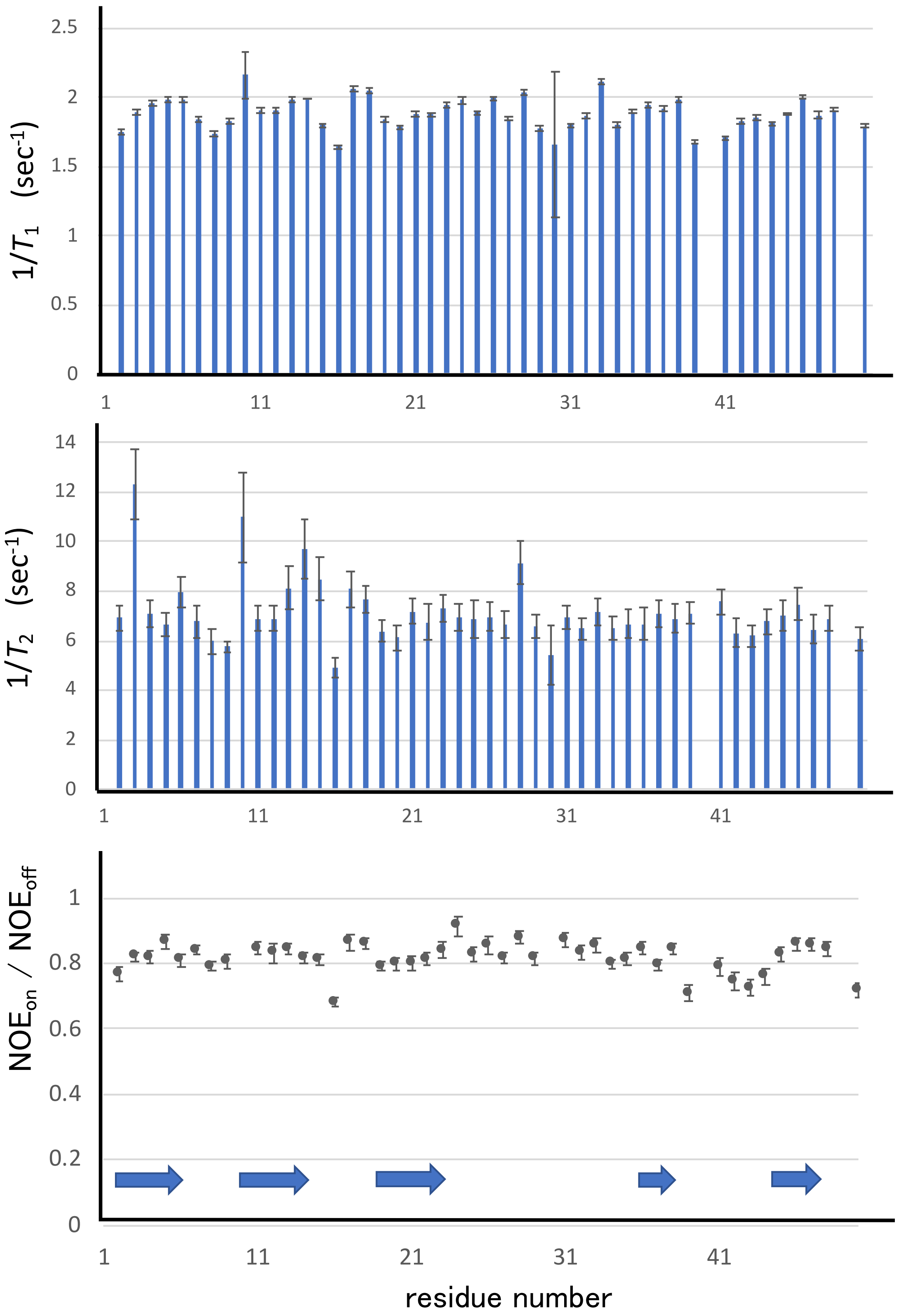

Figure S5. NMR relaxation data of SIB1. $1/T_1$, $1/T_2$, and heteronuclear NOE results are presented. Arrows indicate the β-strand regions.

.
